# Small Bowel Perforation as a Consequence of Strangulated Direct Inguinal Hernia

**DOI:** 10.7759/cureus.12181

**Published:** 2020-12-20

**Authors:** Sherif Monib, Ahmed Hamad, Hany F Habashy

**Affiliations:** 1 Breast Surgery, West Hertfordshire Hospitals NHS Trust, St. Albans and Watford General Hospitals, London, GBR; 2 Breast Surgery, University Hospitals of Derby & Burton, Derby, GBR; 3 Surgical Oncology, Faculty of Medicine Fayoum University, Faiyum, EGY

**Keywords:** groin, hernia, strangulated, bowel perforation, inguinal hernia

## Abstract

Inguinal hernia is probably one of the most common surgical conditions, with strangulation accounting for a good number of acute surgical admissions. It has always been known that direct hernias are less likely to strangulate due to wide hernial defects in comparison to indirect hernia. For that reason, some surgeons do not attempt repair of direct hernias in elderly patients. We present a relatively uncommon case of a 58-year-old gentleman who presented with clinical signs of an incarcerated inguinal hernia; which was found at exploration to be a strangulated direct hernia with small bowel perforation. We believe that direct inguinal hernia should always be included in the differential diagnosis of incarcerated or strangulated groin hernia.

## Introduction

Inguinal hernia repair is probably one of the most common procedures in general surgery. Yet, sometimes it can be a diagnostic challenge a fact that can be attributed to the complex anatomy of the groin. It has always been believed that direct hernias are less likely to present with strangulation due to the relatively wide neck in comparison to indirect inguinal hernia. Based on that, some surgeons do not attempt to direct inguinal hernia repair in older patients. However, one study showed that age is the most important prognostic factor for strangulation, with increased incidence in older patients; they also found that the average diameter of the transversalis fascia hernia defect was 23.8 mm. Hence, they concluded that the diameter of the hernial defect is not directly related to the incidence of strangulation [1].

## Case presentation

**Figure 1:**
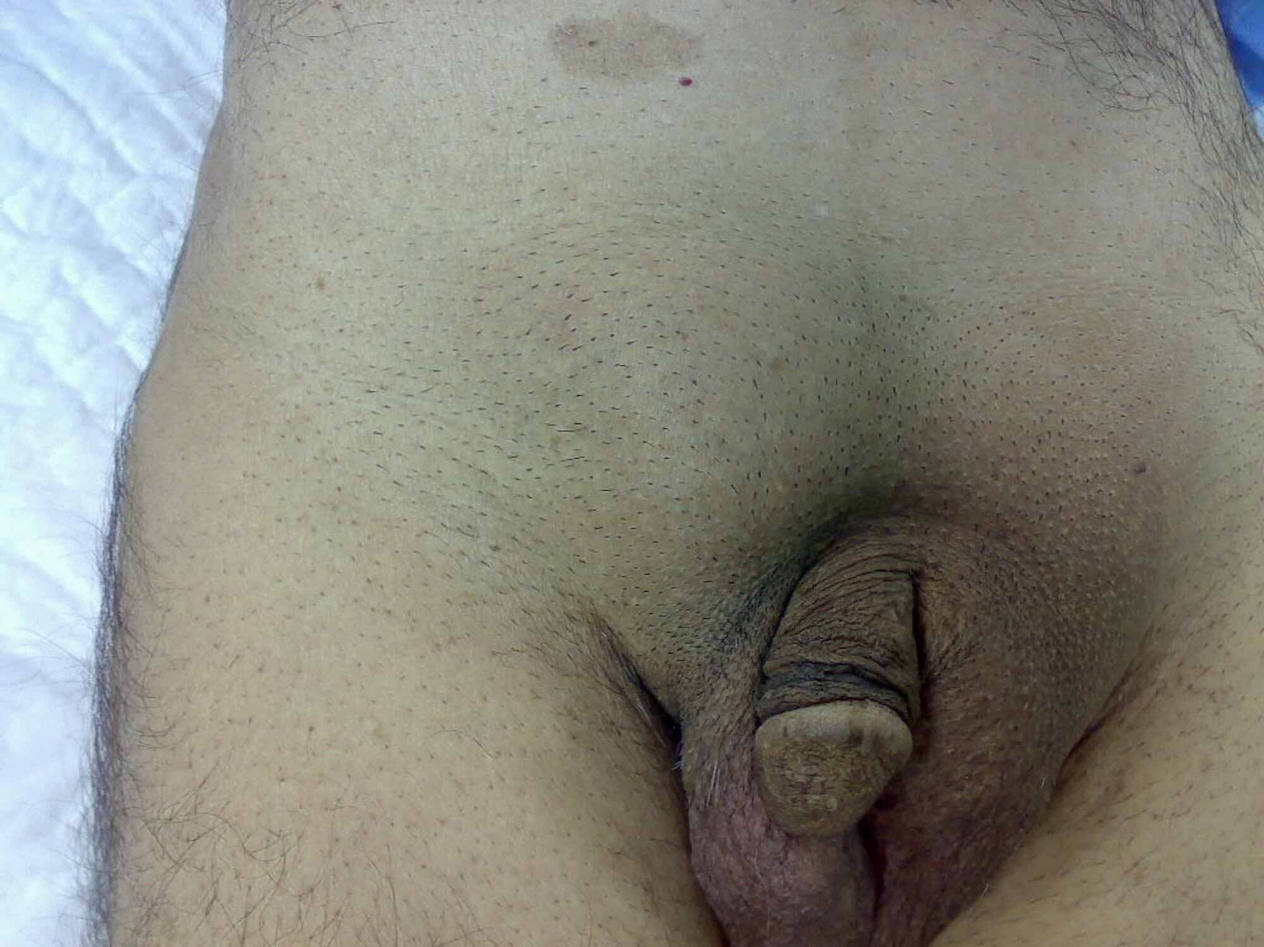
Preoperative photo showing the right groin swelling.

We are presenting a case of a 58-year-old gentleman who presented to the accident and emergency department with a tender right groin swelling of about 36-hour duration, following heavy object lifting, pain was dull aching in character and progressively increasing in intensity. His past medical history included that he was a heavy smoker who smoked about 20 cigarettes per day for about 20 years, and he has been suffering from right groin pain every time he lifted or pushed any heavy object with no other medical conditions of significant importance. General examination was unremarkable; his vital signs were within normal apart from tachycardia of 90 bpm. Abdominal examination revealed lax abdomen with no palpable masses or organomegaly, a tender right direct/indirect inguinal hernia, with no impulse on cough, scrotal examination was normal (Figure 1).

**Figure 2:**
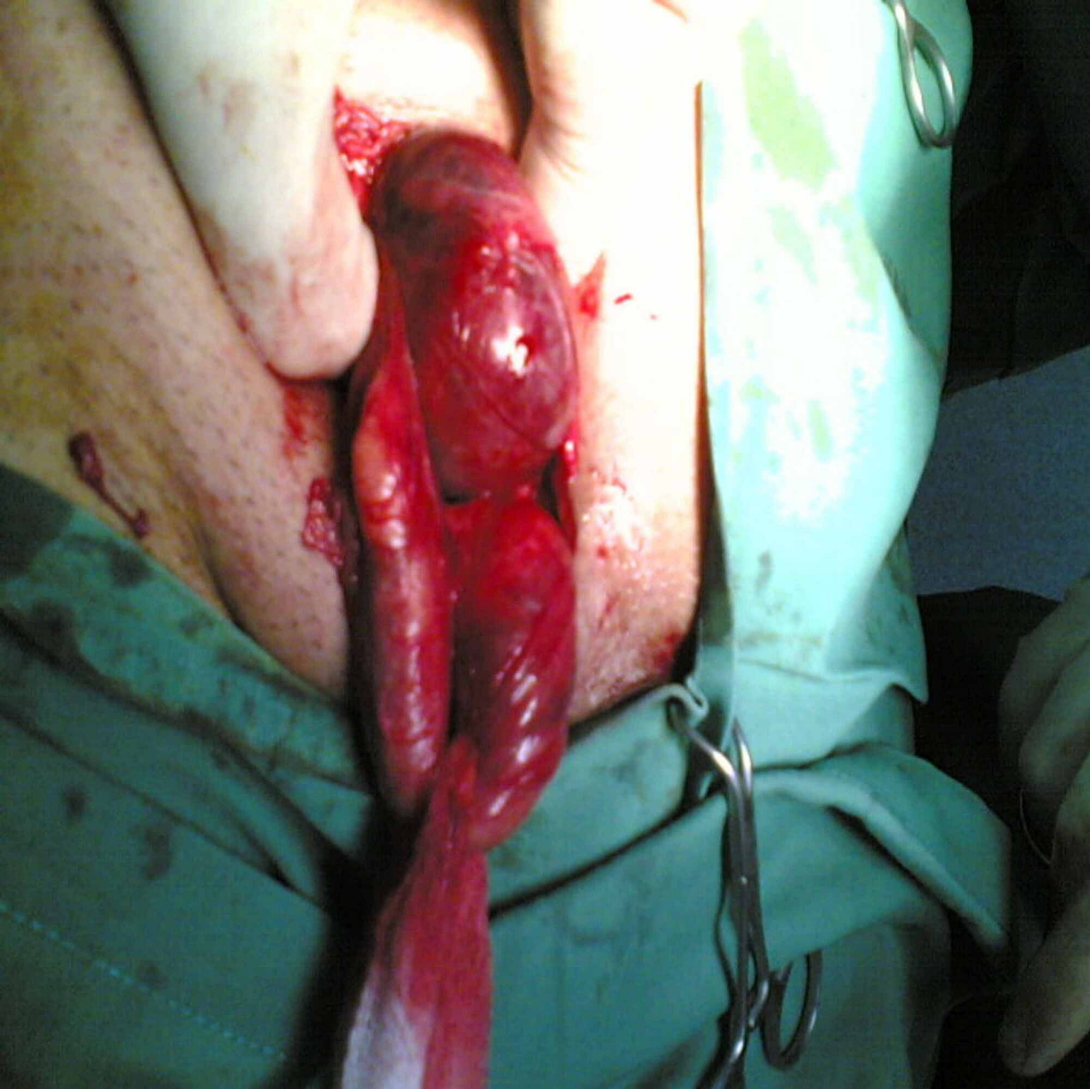
Intraoperative photo showing the direct hernia.

**Figure 3:**
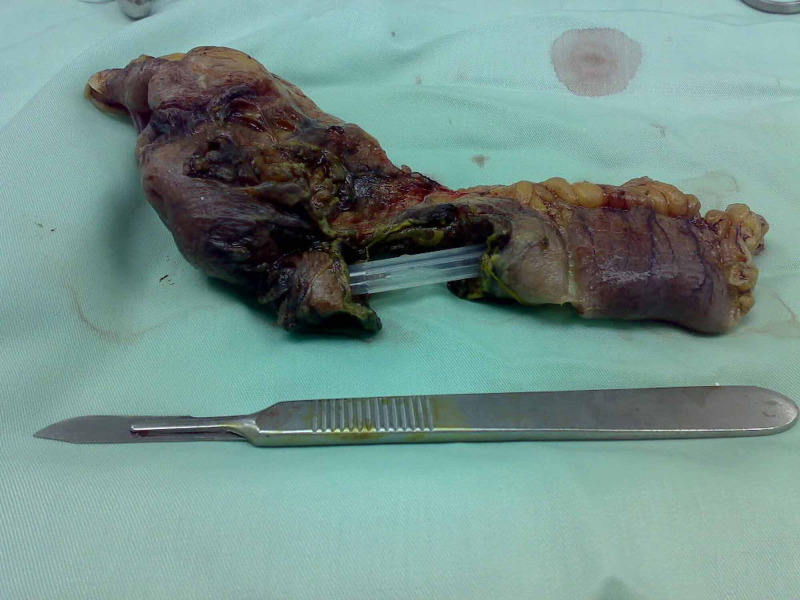
Surgical specimen showing the site of the perforated small bowel.

All his blood were within normal range; no scans were done at that time. The provisional diagnosis was an incarcerated inguinal hernia, so he was consented and taken to the theatre for repair of the inguinal hernia. At operation, it was found to be a strangulated direct hernia containing a perforated loop of the terminal ileum (Figures 2, 3).

 

**Figure 4:**
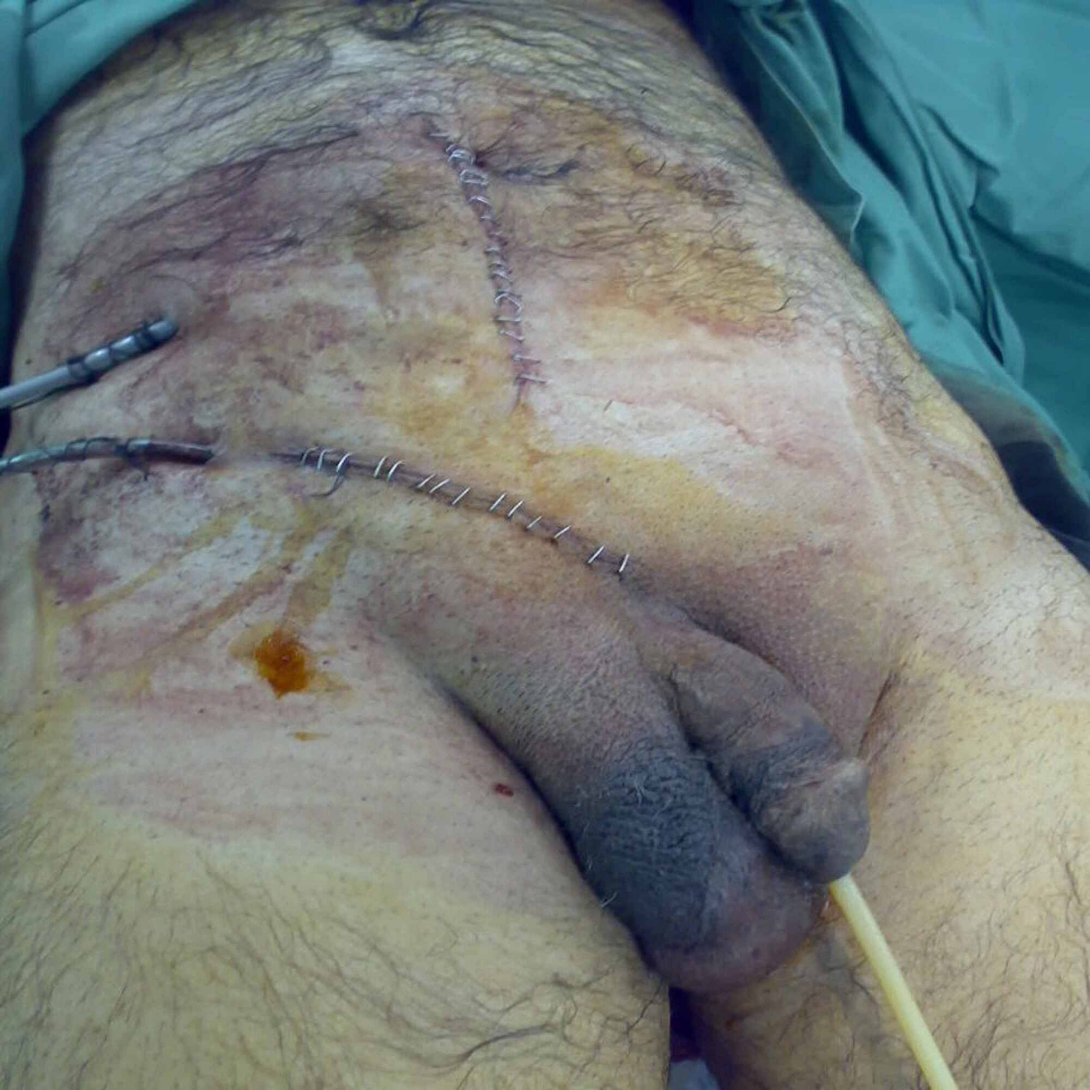
Postoperative picture showing the right groin exploration incision as well as the laparotomy incision.

A laparotomy was carried out through an infra-umbilical midline incision, revealing perforated distal ileum, resection-anastomosis of the perforated segment of the bowel, peritoneal lavage was carried out, two Robinson drains were left in the peritoneal cavity, and the hernia was repaired by Lichtenstein technique (Figure 4).

The patient passed through a smooth postoperative period; he was discharged home on the fifth postoperative day and seen in the clinic after six weeks with no complications.

## Discussion

While inguinal hernias are relatively common, with an estimated prevalence of 6% [2]; incarceration of the hernia only occurs in 10% of cases. Unfortunately, in some cases, incarceration can progress to intestinal obstruction, strangulation, infarction and subsequently perforation, which can lead to unfavourable outcome if not diagnosed and managed promptly [3,4].

Incidence of strangulation varies from 3.25 to 7.16 per 100,000 population per annum [5]. When Gallegos et al. calculated strangulation incidence, based on lengths of medical history from onset of symptoms to diagnosis of strangulation they found that strangulation incidence raises with time from 2.8% at three months to 8.6% at five years [6]. On the contrary, Nyhus et al. noted that 10% to 20% of patients present with a strangulated inguinal hernia as the first presentation [7]. Also, McEntee et al. reported that one-third of patients usually present with manifestations of strangulation only a few days after discovering the presence of the hernia [8].

Watkin et al. reported that in their cohort the risk of strangulation was inversely related to the diameter of the hernia defect (the smaller the defect, the higher the risk of strangulation), they also reported that incidence of strangulation was only 3% in direct hernias [5]. On the contrary Kulacoglu et al. found that age is an important prognostic factor for strangulation, as they noted increased incidence of strangulation in patients older than 60 years, in comparison to those younger than 60 years. They also found that the average diameter of the transversalis fascia defect was 23.8 mm, so the diameter of the fascial defect was found to have an impact on the incidence of strangulation. Still, they noted a significant difference in the incidence of strangulation between indirect hernia, which was 32.6% and direct inguinal hernias which was10.3% [1].

When Hair et al. looked into intraoperative findings of patients presenting with strangulation they found that 50% of hernias emerged through an indirect defect, 30% were recurrent hernias, 10% emerged through a pantaloon hernia (having both direct and indirect components), and 10% emerged through a direct hernia [9].

While it is well established that elective inguinal hernia mesh repair is associated with superior results when compared with Bassini repair; there has always been a debate regarding mesh repair in strangulated inguinal due to the fear of increased incidence of infection hernia. This debate was resolve following multiple studies confirming that even in cases with strangulated inguinal hernia, Lichtenstein “tension-free” mesh repair is safe and effective with low postoperative complications and recurrence rates [10,11].

In our case, the preoperative diagnosis was an incarcerated inguinal hernia; we initially thought that strangulation is unlikely due to the absence of systemic manifestations and signs of bowel obstruction and also the fact that strangulation is very rare with direct hernias. At operation, the hernia was found to be a direct one containing a strangulated perforated loop of the terminal ileum, a rather uncommon finding in a direct hernia. Resection anastomosis of the perforated segment of the bowel was carried out with uneventful intraoperative and postoperative courses.

## Conclusions

Inguinal hernia is a common surgical condition accounting for a good number of elective as well as acute surgical admissions; for the experienced surgeon, diagnosis and repair should be straightforward; however, unexpected operative findings may be encountered requiring an expert approach. The urgent need to repair strangulated inguinal hernias should not have a negative impact on adequate preoperative imaging to ascertain an accurate diagnosis preoperatively and rule out concomitant intra-abdominal pathology. We recommend reporting direct inguinal hernia cases to have an extensive database which will allow us to come up with more informative recommendations.
